# Current Trends of Carcinoma: Experience of a Tertiary Care Cancer Center in North India

**DOI:** 10.7759/cureus.15788

**Published:** 2021-06-21

**Authors:** Manjit K Rana, Tushar S Barwal, Uttam Sharma, Richika Bansal, Karuna Singh, Amrit Pal S Rana, Aklank Jain, Utkarshni Khera

**Affiliations:** 1 Pathology and Laboratory Medicine, All India Institute of Medical Sciences, Bathinda, IND; 2 Research, Central University of Punjab, Bathinda, IND; 3 Pathology, Advanced Cancer Institute, Baba Farid University of Health Sciences, Bathinda, IND; 4 Radiation Oncology, Advanced Cancer Institute, Baba Farid University of Health Sciences, Bathinda, IND; 5 Surgery, Guru Gobind Singh Medical College and Hospital, Baba Farid University of Health Sciences, Faridkot, IND; 6 Biochemistry, Central University of Punjab, Bathinda, IND

**Keywords:** carcinoma, histopathological profile, esophagus, head and neck tumors, incidence

## Abstract

Objective

Cancer incidence across the geographical area is mercurial and factors like dietary habits, environment, social structure, genetics govern relative incidence. Malwa region of Punjab is one such geographical area of India speculated to have a higher incidence of cancer. The current analysis was done to assess the occurrence of cancer in the region and to analyze the trends and types of carcinoma with age, gender, site, and histopathological type, and to compare with the trends mentioned in the literature.

Methods

A retrospective analysis was done to collect and collate 2088 cancer patients' pathological records for three years at a tertiary treatment center. The collated data was digitized and used to create tables and histograms.

Result

Of the 2088 cancer cases, the leading cancer site was breast (24.7%) in females, followed by cancer of female genetic tract (18.9%), whereas in males, the most common site involved was head and neck (17.5%) followed by esophagus (10.3%). The leading cancer type for males was squamous cell carcinoma and for females was infiltrating ductal cell carcinoma. Breast carcinoma was most commonly seen cancer (40.5%) followed by female genital tract carcinoma and esophageal carcinoma in female patients. Whereas in males, head and neck carcinoma was most commonly identified (37.5%) followed by the gastrointestinal tract and esophageal carcinoma. This higher incidence may be attributed to better medical facilities, cancer awareness, and novel government schemes.

Conclusion

Based on our comprehensive analysis, we conclude that there was a change in trends of all types of carcinomas in males and females except breast carcinoma, which was seen as the most common carcinoma in female patients. Our findings suggest and support the strong implementation of cancer awareness programs and epidemiological studies to know the changing trends of risk factors in the region.

## Introduction

Despite recent advancements in the curative and therapeutic approaches concerning cancer, it remains the leading cause of mortality in developed countries and the second leading cause of mortality in developing countries [1]. Furthermore, increased economic development in developing countries, coupled with an aging population and adoption of cancer-associated lifestyle is the leading cause of increased cancer incidence. Recent Global Cancer Observatory (GLOBOCAN) data insulated an increasing trend in cancer mortality globally with an incidence of ~19 million and mortality of ~9 million in 2020. Affirming the global incidence patterns, recent GLOBOCAN data have projected an incidence of ~1.3 million and mortality of ~0.8 million for India [2].

Systematic collation of cancer parameters like genetics, dietary habits, environment, and social structure are essential clues to understanding and predicting cancer incidence and assisting in developing a baseline to direct curative approaches [3]. In this regard, initial efforts were made in the year 1982 via formulating population-based cancer registries (PBCRs) and hospital-based cancer registries (HBCRs) under the National Cancer Registry Program (NCRP), National Centre for Disease Informatics and Research (NCDIR) of the Indian Council of Medical Research (ICMR; ICMR- NCDIR-NCRP), Bengaluru, India. To date, several NCRP reports have been published. Furthermore, independent research groups have conducted several studies that describe national-level patterns associated with cancer burden and epidemiology across India [4-9]. Unfortunately, despite employing comprehensive cancer prediction strategies and the disproportionate numbers of health care workers and patients, decentralized population, and geographical constraints, a systemic and complete understanding of the magnitude and time trends in cancer distribution is missing [10].

This study's primary objective is to provide a relevant framework for accessing and predicting the impact of cancer in the state of Punjab, India. Furthermore, the study aims to provide an outline to determine the status and patterns of cancer in the Malwa region of Punjab. Additionally, it will help guide appropriate support for action to strengthen efforts in improving cancer prevention and control.

## Materials and methods

**Figure 1:**
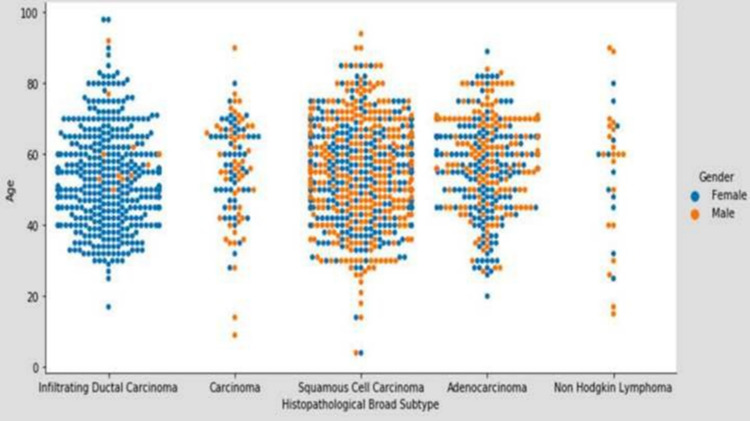
Age-wise distribution of various histopathological broad subtypes

The hospital-based three-year retrospective study was conducted and cancer records were generated from 2016 to 2019 and analyzed using central records. The study comprised 2495 patients who visited the pathology department for a histopathology examination. A detailed gross examination of tissue specimens was done followed by histopathological analysis. Neoplastic lesions were classified according to the recent WHO classification. Based on the primary histopathological analysis, out of the total patients, 407 cases were found to be non-malignant, constituting 13% non-neoplastic, 49.6% benign, 18.6% borderline cases, and 2088 patients with malignant tumors. The majority of the patients with non-neoplastic conditions were of chronic inflammatory pathology only. Benign and borderline lesions were primarily constituted by fibroadenomas and atypical ductal cell hyperplasia, respectively. Further, patients were categorized based on the predominant microscopic pattern of malignancy as depicted in Figure 1.

Immunohistochemical examination of all breast carcinomas and selected cases of unknown primary malignancy was performed. All insights were recorded, tabulated, and depicted graphically.

## Results

The study was conducted for three years and a total of 2495 patients were subjected to histopathology screening. After the initial screening, 2088 patients were considered relevant for the study, and their age, gender, data of histopathological patterns were collected and collated. The majority of the cancer cases involved the breast, head and neck, esophagus, and cancer of the female genital tract, the breast being the leading cancer site.

Age and sex

**Figure 2:**
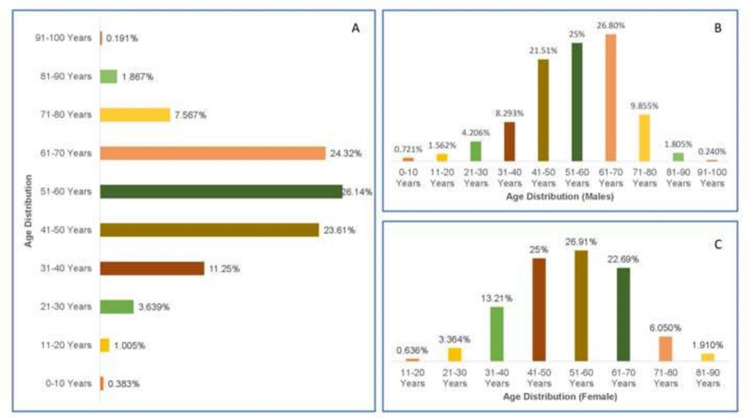
[A] Overall age- and gender-wise distribution of cancer patients, [B] Age-wise incidence of cancer in males, [C] Age-wise incidence of cancer in females

The age and sex distribution of 2088 cancer cases showed most patients between the age group four years to 98 years of age. Peak incidence was between the age of 51 to 60 years in females and 61 to 70 years in males (Figures 2A, 2B, 2C).

**Figure 3:**
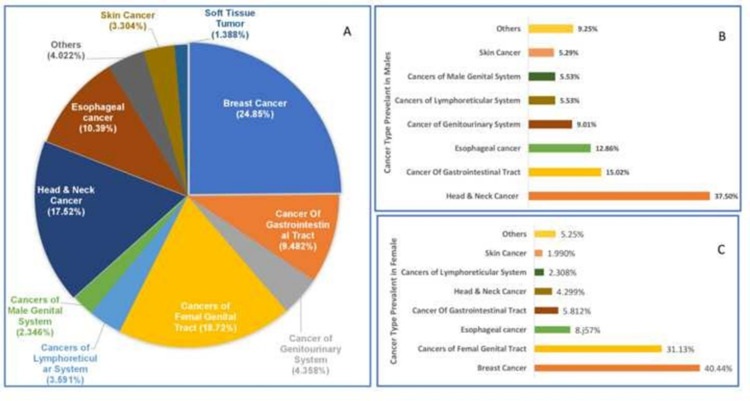
[A] Overall most prevalent cancers, [B] Most prevalent cancer in males, [C] Most prevalent cancer in females

Among the 2088 cancer cases, 60.0% were female and 40.0% were male. Additionally, the top 10 leading sites of cancer in males and females with incidence are depicted in Figures 3A, 3B, 3C.

Breast carcinoma was the most commonly seen cancer (40.5%), followed by female genital tract carcinoma and esophageal carcinoma in female patients. Whereas in males, head and neck carcinoma was the most commonly identified (37.5%), followed by the gastrointestinal tract and esophageal carcinoma.

Histopathological pattern

Overall histopathological evaluation of 2088 cancers revealed squamous cell carcinoma to be the most prevalent histopathological type (37.93%); furthermore, the prevalence of squamous cell carcinoma is more in males (52.64%) as compared to females (28.18%) patients. Based on our analysis, infiltrating ductal carcinoma (22.36%) was found to be the second most prevalent histopathological subtype with a very high incidence in females (36.30%). Microscopic examination of breast tumors revealed 80.6% malignant tumors (Table 1).

**Table 1:** Histopathological pattern of carcinoma breast

Sr. No.	Microscopic type	Percentage
1.	Infiltrating ductal cell carcinoma -Not otherwise specified	93.8%
2.	Infiltrating ductal cell carcinoma -Mixed type	1.8%
3.	Infiltrating lobular carcinoma	1.6%
4.	Invasive papillary carcinoma	1.4%
5.	Micropapillary carcinoma	1.2%
6.	Others (Paget’s disease, Mucinous carcinoma, Tubular Carcinoma, Metaplastic carcinoma, Medullary carcinoma, Inflammatory carcinoma)	0.2%

Microscopic examination of head and neck tumors (oral cavity, nasopharynx, larynx, and ear only) revealed 81.5% malignant tumors. Microscopic examination of esophageal tumors revealed 96.5% malignant tumors, constituting 49.3% squamous cell carcinoma moderately differentiated (SCC MD), 23.7% squamous cell carcinoma poorly differentiated (SCC PD), 6.2% squamous cell carcinoma well-differentiated (SCC WD), 1.7% adenocarcinoma, and 18.8% others. Microscopic examination of gastrointestinal tumors (common site being rectum, 53%) revealed 82.5% malignant tumors, constituting 79.3% adenocarcinoma (64.6% MD, 18% PD, 17.3% WD), 3.1% SCC, and 17.6% others. Microscopic examination of female genital tract tumors revealed 80.8% malignant tumors, constituting 58.7% cervical cancer (87.1% SCC, 11.0% adenocarcinoma), 10.7% endometrial carcinoma, 24.2% ovarian carcinoma, and 6.4% others. Microscopic examination of other tumors revealed as depicted in Table 2.

**Table 2:** Organ-wise histopathological patterns SCC: squamous cell carcinoma

Sr. No.	Organ/ System	Predominant Microscopic Pattern (%)
1.	Central Nervous System	Glioblastoma Multiforme IV (52%)
2.	Eye	Neuroblastoma/ Retinoblastoma (50% / 50%)
3.	Skin	Basal cell carcinoma (32.2 %), SCC (32.2%)
4.	Genitourinary system	Adenocarcinoma prostate (59.1%) (Males), Renal Cell Carcinoma (90%) Females
5.	Lung	Adenocarcinoma (52.6%)
6.	Lymphadenopathy	Non-Hodgkin’s Lymphoma (45.4%), Metastatic carcinomatous deposits of unknown primary (27.2%)
7.	Male genital system	SCC penis (75%)
8.	Soft tissue	Spindle cell sarcoma (86.2 %)
9.	Thyroid	Papillary carcinoma (81.2%)
10.	Salivary gland	Mucoepidermoid carcinoma (50%)
11.	Retroperitoneum	Liposarcoma (66.6%)

## Discussion

Cancer incidence pattern varies from country to country, and in a geographically distinct country like India, the incidence pattern differs from one region to another. Furthermore, developing countries like India tend to have a lower cancer incidence of approximately 100/100,000 compared with about 361/100,000 in the USA. The main reason for this disproportionate incidence is higher mortality due to infectious disease in developed countries than in developing countries; additionally, as aging increases, the chances of developing cancer also increase [11].

The present study is a retrospective analysis depicting prominent cancer types, gender distribution, age distribution, and foremost histopathological subtypes. Based on our research, cancer incidence in females (60.00%) was much higher than in males (40.00%). Estrogen plays an important role in the chances of higher incidence as well as presentation of carcinoma at an early age in females. This is in alignment with various previous studies conducted in Malwa areas by several groups like Bal et al. (2015) [male 35%; female 65%], Thakur et al. (2008) [male 25.2%; female 74.7%] and Aggrawal et al. (2015) [male 39.1%; female 60.9%] [3,12,13]. With a varying sample size, each study validated an increased cancer incidence in females compared to male patients. Inconsistent with the above findings, Sandhya et al. (2009) reported a higher cancer incidence in males (53.99%) when compared to females (40.01%) in Nellore district Andhra Pradesh. The contrasting gender distribution proves the importance of geographical location in the altered incidence of cancer amongst the population; furthermore, a higher incidence of cancer in males of Nellore District Andhra Pradesh might be attributed to dietary and social habits like higher consumption of spices leading to increased incidence of gastric cancer and consumption of tobacco and betel nut leading to increased oral cancer amongst males. However worldwide data showed a higher incidence rate for all cancers in men at 19% (222 per 100,000) than in women (186 per 100,000) in 2020 [14].

Our study's most affected female group was 51-60 years of age (26.91%) followed by 41-50 years age group with an incidence of (25.00%). Contrary to the above, the male's most affected age group was 61-70 years (26.80%) followed by 51 to 60 years (25%). These results were quite similar to the study performed by Bal et al. (2015), in which the most affected age group of females was 50-54 years of age group, with (11.2%) incidence, followed by 60-64 years of age group incidence 10.8%. Similarly, males in the age group 65-69 with a 5.6% incidence, followed by 50-54 years age group with an incidence of 4.8% [3].

Similarly, overall, the most prevalent cancer in female was breast cancer (40.44%), followed by cancer of the female genital tract (31.13%), which included cervical cancer (58.31%), ovarian cancer (23.52%), endometrial cancer (11.50%), and others (6.00%). Additionally, in males, the leading cancer type was head and neck cancer (37.50%), followed by cancer of the gastrointestinal tract (15.02%) and oesophageal cancer (12.86%). Out of 15.02%, cases of gastrointestinal tract (GIT) carcinoma, colon cancer were seen in 60.3% of the cases, followed by carcinoma stomach in 14.8% of the cases. 

According to GLOBOCAN 2020, the most common carcinoma in females was breast cancer (11.7%) as seen in the current analysis followed by lung carcinoma (11.4%) with carcinoma cervix (3.1%) at the eighth position. And in males, the most common cancer was lung carcinoma (14.3%), followed by prostate (14.1%) and GIT (10.6%), with the esophagus at seventh (4.2%) position [2]. However, according to the previous studies were done by Bal et al. (2015) and Sandhya et al. (2009), the most prevalent cancer was breast cancer (26.8% | 30.3%) followed by cervical cancer (13%) in females [3,14]. In males, the most pervasive cancer was colon cancer (3.6%), followed by esophageal cancer (2.6%) [3]. These findings suggest a strict implementation of screening programs and cervical cancer awareness programs in Punjab. As per the report of NCRP 2016 and GLOBOCAN 2020, an extraordinary increase in head and neck carcinoma was detected in the Indian population. Our data also showed head and neck cancer as the most common carcinoma in the region, so further larger epidemiologic studies are need of the hour to rule out the risk factors in the area [2].

Carcinoma breast shows diversity in histopathological types that are detected on the basis of cytomorphological architecture only. Different presentations and prognoses recommend clinical follow-ups and genetic evaluation for a better understanding of this lesion [15,16]. The most common histopathological type was SCC WD among head and neck tumors (oral cavity, nasopharynx, larynx only) followed by others, these findings were more or less similar to the previous studies done [17].

In concordance with the literature, the most common microscopic type of esophageal tumors was constituted 49.3% SCC MD, 23.7% SCC PD, 6.2% SCC WD, followed by 1.7% adenocarcinoma and 18.8 % others in our experience also [18]. Though a male predominance in the incidence of adenocarcinoma has been noted worldwide, in our study, relatively lesser cases were seen in males with a male-to-female ratio of 1:3 [19]. Rectal adenocarcinoma (52%) was seen more prevalent in our region with microscopy revealing 64.6% MD type. As per Rana S, gastric cancer is a more prominent problem in northeastern and southern states of the Indian subcontinent [20]. The prevalence of cervical cancer among gynecological malignancies was 58.7% constituting 87.1% SCC and 11.0% adenocarcinoma. Kumari A et al. also observed cervical carcinoma as the most prevalent cancer category with SCC being the commonest histopathological type [21].

Tumors of the central nervous system (CNS) constitute approximately 2% of all malignancies. A study conducted by AlmutrafiIn A et al. showed medulloblastoma as the first common malignant lesion of the CNS [22]. Jain A and fellows had observed astrocytoma (34.7%) as the most common CNS tumor in children in India [23]. Another study, done by Madhavan R, also showed astrocytoma (52%) as the most common malignancy of CNS tumors. However, a study done by Jaiswal J et al. mentioned meningiomas (23.2%) as the first common CNS tumor followed by glioblastomas (15.5%) in adults. Being a referral institute for radiotherapy in the region, in contrast to previous regional and international studies, the glioblastoma multiforme grade IV was more frequent in the current analysis [24,25].

Among children, retinoblastoma is the most common primary intraocular malignancy. Although it is curable in the early stages, the majority of the patients in India present in the late stages as seen in our experience. Enucleation was done after chemotherapy treatment in all the cases [26].

Amongst skin carcinomas, both basal cell carcinoma (BCC) and SCC showed equal frequency. Though many studies were done in the past showed a variable incidence of BCC and SCC in skin carcinoma [27].

Prostate cancer is increasing worldwide and was the fourth common carcinoma in males in the current analysis. Increased incidence has been considered due to increased screening of serum prostate-specific antigen and increased numbers of biopsy cores taken. In our study, based on age and prostate volume, 6-12 cores of the biopsy were taken and adenocarcinoma prostate was seen most common carcinoma amongst the genitourinary system in males [28-30]. In contrast to previous studies done in the past, renal cell carcinoma was more commonly seen in females with a female-to-male ratio of 1.5:1 [31]. The incidence of lung adenocarcinoma has been increasing gradually and displacing SCC. Our findings were similar to the trends [32,33]. Inconsistent with the worldwide increasing trends of non-Hodgkin’s lymphoma (NHL), NHL was seen as a more common disorder among all lymphadenopathies. Among genital tumors, carcinoma penis constituted 75% of cases [34,35].

In a study done by Bajpai J et al., synovial sarcoma was the most common histopathological type of sarcoma, whereas spindle cell sarcoma NOS (not otherwise specified) was the commonest type in our case [36]. Among thyroid carcinomas and salivary gland carcinomas, papillary carcinoma thyroid and mucoepidermoid carcinoma were the most commonly reported variants [34-38]. Primary retroperitoneal sarcomas are relatively uncommon tumors, liposarcoma being most frequent and presenting difficulty in diagnosis and resection due to larger sizes [39].

Limitations of the study

Population-based studies are essential to validate the magnitude and pattern of cancer in an area. In the present hospital-based study, we have included self-reported cases to the hospital for diagnosis and treatment. Patients unable to recognize an illness or unable to bear financial implications associated with cancer treatment were missed in such a scenario. Furthermore, the relative incidence of a specific cancer type in a hospital depends on the presence of the particular diagnostic and treatment facility, the popularity of the treating physician, and the affordability of treatment. 

## Conclusions

The hospital-based study indicated cancer profiles for the southern region of Punjab, India. Out of the total 2495 microscopically diagnosed patients who visited the pathology department for histopathology verification, 2088 patients were considered relevant for this study. Overall, breast cancer was the most prevalent cancer amongst all cancers. The most prevalent cancer in males was head and neck cancer. The maximum number of cancer cases were observed in the age group 51-60 years of age. The youngest cancer patient was a four-year-old girl suffering from retinoblastoma. The oldest cancer patient was a 98-year-old female suffering from breast cancer. The most prevalent histopathological subtype was squamous cell carcinoma (37.93%) followed by infiltrating ductal carcinoma (22.36%). Our data recommends the need for large site-specific and region-specific studies to conduct and research demonstrating cancer incidence, cancer profile, and risk factors associated with cancers.
